# β-Boswellic Acid Suppresses Migration and Invasion and Attenuates TGF-β1-Induced EMT-Related Changes in PC-3 Prostate Cancer Cells

**DOI:** 10.3390/cimb48101069

**Published:** 2026-10-16

**Authors:** Kaixin Song, Kunpeng Zang, Zihan Yang, Zhirong Tan, Xiaohui Geng, Jiale Wang, Lixi Zhu, Sijie He, Sian Pan

**Affiliations:** 1 School of Pharmacy, Hunan University of Chinese Medicine, Changsha 410208, China; skx759959@stu.hnucm.edu.cn (K.S.); 202530867@stu.hnucm.edu.cn (K.Z.); 202304060137@stu.hnucm.edu.cn (Z.Y.); 202530864@stu.hnucm.edu.cn (X.G.); 2 College of Integrated Traditional Chinese and Western Medicine, Hunan University of Chinese Medicine, Changsha 410208, China; 202407010721@stu.hnucm.edu.cn; 3 College of Traditional Chinese Medicine, Hunan University of Chinese Medicine, Changsha 410208, China; 202402130136@stu.hnucm.edu.cn (J.W.); zzjzns0205@stu.hnucm.edu.cn (L.Z.); 202402130113@stu.hnucm.edu.cn (S.H.)

**Keywords:** β-boswellic acid, prostate cancer, PC-3 cells, epithelial–mesenchymal transition, migration, invasion, multi-omics, machine learning

## Abstract

β-Boswellic acid (BA) has potential antitumor activity, but its effects on migration, invasion, and epithelial–mesenchymal transition (EMT)-related phenotypes in prostate cancer cells remain unclear. In this study, we integrated public transcriptomic data, candidate molecule prediction, machine learning, functional association analysis, and molecular simulations with MTT, wound-healing, Transwell, qPCR, Western blotting, transcriptomic analysis, and data-independent acquisition proteomics to evaluate BA effects on PC-3 cells and explore its potential molecular basis. We identified 22 candidate BA-related molecules and prioritised DPP4, MME, and ABL1 for further analysis. Higher BA concentrations reduced PC-3 cell viability. At the same time, BA suppressed wound closure, migration, and invasion and attenuated TGF-β1-induced EMT-related changes, accompanied by increased E-cadherin and decreased N-cadherin, Snail, and ZEB1 levels. DPP4, MME, and ABL1 also exhibited BA-associated expression changes. Integrated transcriptomic and proteomic analyses suggested that BA-associated molecular alterations involved the cell cycle, DNA replication, cell adhesion, cytoskeletal remodelling, and TGF-β-related processes. In conclusion, BA suppresses migration and invasion in PC-3 cells and attenuates TGF-β1-induced EMT-related changes. Candidate molecules including DPP4, MME, and ABL1, together with the multi-omics findings, provide exploratory clues for further investigation of the potential molecular basis of BA activity.

## 1. Introduction

Prostate cancer (PCa) is one of the most common malignancies in men and a leading cause of cancer-related mortality among men worldwide [1,2]. Although some prostate cancers progress relatively slowly, a subset of patients develop highly invasive and metastatic disease. Bone metastasis, in particular, is closely associated with advanced disease progression and poor prognosis [3,4,5,6]. Androgen deprivation therapy (ADT) remains an important treatment option for metastatic prostate cancer. However, as the disease progresses, some patients develop castration-resistant prostate cancer (CRPC) and face metastasis and treatment resistance [7,8,9]. Therefore, identifying candidate agents that suppress malignant phenotypes such as migration and invasion remains an important research objective.

Epithelial–mesenchymal transition (EMT) is a biological process closely associated with tumour cell migration, invasion, metastasis, and treatment resistance and has received considerable attention in prostate cancer progression [10,11,12,13,14,15]. EMT typically involves the loss of epithelial characteristics, the acquisition of mesenchymal features, and changes in associated transcriptional programmes. These changes include decreased E-cadherin expression and increased expression of mesenchymal markers such as N-cadherin and vimentin, thereby enhancing tumour cell motility and invasive capacity [12,16,17,18]. Previous studies have shown that TGF-β, PI3K/Akt, and other signalling networks regulate EMT and phenotypic plasticity in prostate cancer cells [19,20,21,22,23,24]. Therefore, evaluating the effects of naturally derived bioactive small molecules on EMT-related migration and invasion, and identifying candidate molecules associated with these changes, may provide new insights into potential intervention strategies for prostate cancer.

Natural products and their derivatives have long been important sources of lead compounds for drug discovery and the treatment of various diseases, including cancer [25,26]. β-Boswellic acid (BA) is a naturally occurring pentacyclic triterpenoid derived from the resin of Boswellia serrata. Boswellic acids exhibit various pharmacological activities, including anti-inflammatory and potential antitumour effects [27,28,29]. Previous studies have suggested that certain boswellic acid derivatives can inhibit prostate cancer cell growth and survival and promote apoptosis-related processes, with reported involvement of IKK/NF-κB and Akt/STAT3 signalling [30,31]. However, existing studies have mainly focused on the antiproliferative and pro-apoptotic effects of selected boswellic acid derivatives. The effects of BA on EMT-related migration and invasion in prostate cancer cells remain insufficiently investigated, and the molecular changes potentially associated with these effects require further clarification.

Based on this background, the present study aimed to evaluate the effects of BA on migration, invasion, and EMT-related phenotypes in prostate cancer cells and to explore candidate molecules potentially associated with these effects. We first integrated public transcriptomic data, target prediction, and machine learning approaches to screen and prioritise candidate molecules, followed by molecular simulations for complementary computational evaluation. We then examined the effects of BA on cell viability, migration, invasion, and EMT-related molecular expression in PC-3 cells. We also used a TGF-β1-induced model to further evaluate BA-related EMT changes. In addition, transcriptomic and proteomic analyses were performed to characterise global expression changes and associated biological processes following BA treatment. This study provides experimental evidence for further understanding the biological effects of BA in prostate cancer and its potential molecular basis.

## 2. Materials and Methods

### 2.1. Reagents, Cell Culture, and Treatment

β-Boswellic acid (BA; CAS No. 631-69-6; purity ≥98%) was purchased from Bio Cuiran Biotech Co., Ltd. (Wuhan, China; Cat. No. CRN0049) and dissolved in dimethyl sulfoxide (DMSO) to prepare a 150 mM stock solution. The stock was then diluted with culture medium to the indicated concentrations before use. Recombinant human TGF-β1 was purchased from MedChemExpress (Shanghai, China; Cat. No. HY-P70543), and docetaxel was purchased from Solarbio (Beijing, China; Cat. No. ID0400).

Human PC-3 prostate cancer cells were obtained from Procell Life Science & Technology Co., Ltd. (Wuhan, China; Cat. No. CL-0185) and cultured in RPMI 1640 medium (Meilunbio, Dalian, China; Cat. No. PWL004) supplemented with 10% fetal bovine serum and 1% penicillin–streptomycin at 37 °C with 5% CO_2_. Cells in the logarithmic growth phase were used for experiments, and mycoplasma testing was performed regularly. Details of the manufacturers, locations, and catalogue or model numbers of other reagents, consumables, and equipment are provided in Supplementary Table S1.

### 2.2. Collection, Preprocessing, and Differential Expression Analysis of Public Transcriptomic Data

Prostate cancer transcriptomic data were obtained from the NCBI Gene Expression Omnibus (GEO) database. GSE3325, GSE6956, and GSE16120 formed the discovery cohort, comprising 40 non-tumour control samples and 133 prostate cancer samples. GSE46602, GSE55945, and GSE200879 formed the external validation cohorts, comprising 31 non-tumour control samples and 177 prostate cancer samples. After probe annotation and gene symbol standardisation, the datasets were processed. When multiple probes mapped to the same gene, expression values were averaged. Batch effects in the discovery cohort were corrected using the ComBat method in the sva (version 3.54.0) package, and principal component analysis (PCA) [32] assessed the effectiveness of batch correction. Differential expression analysis was then performed using the limma (version 3.62.2) package, with FDR < 0.05 and |log2FC| > 0.585 as the screening thresholds [33]. The external validation cohorts were used to evaluate candidate model performance and validate the expression patterns of candidate molecules.

### 2.3. Prediction and Screening of β-Boswellic-Acid-Related Candidate Molecules

Potential BA-related proteins were predicted using SwissTargetPrediction and PharmMapper, with the species restricted to Homo sapiens on both platforms [34,35]. We retained predictions with a probability > 0 from SwissTargetPrediction, then merged the results from both platforms, deduplicated them, and converted them to standard gene symbols. These genes were intersected with differentially expressed genes identified in prostate cancer to obtain candidate BA-related molecules. This intersection was used only to screen candidate molecules and did not establish direct pharmacological interactions between BA and the corresponding proteins.

### 2.4. Machine Learning Modeling and Prioritization of Candidate Molecules

To further screen and prioritise candidate BA-related molecules, we constructed machine learning models using the expression matrix of 22 candidate molecules from the discovery cohort. We included Lasso, Support Vector Machine (SVM), Random Forest (RF), glmBoost, Stepglm, Ridge, Elastic Net, Gradient Boosting Machine (GBM), Linear Discriminant Analysis (LDA), XGBoost, and Naive Bayes, yielding 127 candidate models in total. Model performance was evaluated using stratified five-fold cross-validation. Models with favourable overall performance were selected based on AUC values in the discovery and independent external validation cohorts.

Following a comprehensive comparison, we selected the glmBoost + Elastic Net model (alpha = 0.2), and its retained features were included in further analyses. We then evaluated these features using a Random Forest model and applied SHapley Additive exPlanations (SHAP) to quantify each feature’s relative contribution to the model output. Feature importance was ranked according to the mean absolute SHAP values [36,37,38]. These machine learning analyses were used solely to screen and prioritise candidate BA-related molecules. They were not intended to establish a clinical diagnostic model or to provide evidence of direct binding or causal pharmacological effects between BA and the corresponding proteins.

### 2.5. Functional Enrichment and Functional Association Network Analysis

A functional association network of candidate BA-related molecules was constructed using the GeneMANIA platform to explore potential functional relationships among the candidates in relation to BA. Gene Ontology (GO) and Kyoto Encyclopedia of Genes and Genomes (KEGG) enrichment analyses were then performed using the clusterProfiler (version 4.14.6) package [39,40] to examine BA-associated biological functions. GO analysis included three categories: biological process (BP), cellular component (CC), and molecular function (MF). The Benjamini–Hochberg method was used for multiple-testing correction, and an adjusted *p*-value < 0.05 was considered statistically significant. These enrichment and network analyses were used to characterise BA-associated biological processes and functional associations involving the candidate molecules, rather than to establish direct regulation of the corresponding pathways or molecular networks by BA.

### 2.6. Molecular Docking and Molecular Dynamics Simulation

Based on candidate molecule screening, machine-learning-based prioritisation, and functional association analysis, DPP4, MME, and ABL1 were selected as the main candidate BA-related proteins for docking analysis. The three-dimensional structure of BA was obtained from PubChem, and protein structures were retrieved from the Protein Data Bank (PDB). Receptor proteins were prepared by removing water molecules and co-crystallised small molecules, followed by hydrogen addition and charge assignment. Molecular docking was then performed using AutoDock Vina (version 1.2.3), and potential interactions were evaluated based on predicted binding energies and docking conformations [41].

Molecular dynamics simulations were subsequently performed for 100 ns on the BA–DPP4, BA–MME, and BA–ABL1 complexes [42]. Each system underwent energy minimisation, followed by NVT and NPT equilibration before the production run. The dynamic conformational characteristics of the complexes were evaluated using RMSD, RMSF, radius of gyration (Rg), solvent-accessible surface area (SASA), and the number of hydrogen bonds. Binding free energies were calculated using the MM/PBSA method [43]. Three independent simulations were performed for each system. These computational analyses were used to assess the candidate protein complexes and were not considered experimental evidence of direct binding.

### 2.7. Cell Viability Assay

The effects of BA on PC-3 cell viability were evaluated using the MTT assay [44]. PC-3 cells were seeded in 96-well plates and cultured for 24 h before treatment with different concentrations of BA (20–200 μM) for 24, 48, or 72 h. This concentration range was selected to evaluate the concentration-dependent effects of BA on cell viability and estimate IC_50_ values at different treatment durations. The experimental groups included an untreated control group (Control), a vehicle control group (Vehicle), and BA-treated groups. The Vehicle group received DMSO at the same final concentration as that in the highest BA concentration group (200 μM). After treatment, MTT solution was added, and the cells were incubated further. The culture medium was then removed, and the resulting formazan crystals were dissolved in DMSO before absorbance was measured. Relative cell viability was calculated using the untreated control group as the reference. IC_50_ values were determined by nonlinear regression analysis using GraphPad Prism. Technical replicates were included for each treatment condition, and all experiments were independently repeated at least three times. The MTT assay primarily reflects cellular metabolic activity and was used as an indirect measure of cell viability [45].

### 2.8. Wound-Healing Assay

A wound-healing assay was performed to evaluate wound closure in PC-3 cells under different treatment conditions [46]. PC-3 cells were seeded in 6-well plates and cultured until nearly confluent. A scratch was then made in the cell monolayer using a sterile pipette tip, and the cells were gently washed with PBS to remove detached cells. The medium was replaced with serum-free RPMI 1640 medium containing the indicated treatments. The experimental groups included a control group, BA-treated groups (15, 25, and 35 μM), a docetaxel positive control group (20 nM), a TGF-β1 group (5 ng/mL), and a TGF-β1 + BA group (5 ng/mL TGF-β1 + 25 μM BA).

Images of the same areas were captured at 0, 6, 12, and 24 h after treatment, and wound areas were measured using ImageJ (version 1.54) software. The wound closure rate was calculated as follows: wound closure (%) = [(A_0_ − A_t_)/A_0_] × 100%, where A_0_ represents the wound area at 0 h and A_t_ represents the wound area at the corresponding time point. Each experiment was independently repeated at least three times. Scale bars in representative microscopic images indicate 1000 μm.

### 2.9. Transwell Migration and Invasion Assays

Transwell assays were performed to evaluate PC-3 cell migration and invasion under different treatment conditions [47]. The experimental groups included a control group, BA-treated groups (15, 25, and 35 μM), a docetaxel positive control group (20 nM), a TGF-β1 group (5 ng/mL), and a TGF-β1 + BA group (5 ng/mL TGF-β1 + 25 μM BA). All BA concentrations were below the IC_50_ determined by the 48 h MTT assay to minimise the potential influence of cytotoxicity on the assessment of cellular phenotypes.

Following the indicated treatments, cells were seeded into the upper chambers of the Transwell inserts, while serum-containing medium was added to the lower chambers as a chemoattractant. After 24 h of incubation, cells that had not migrated or invaded through the membrane were gently removed from the upper surface. Cells that had passed through the membrane and adhered to its lower surface were fixed and stained with crystal violet. Images were captured from multiple randomly selected, non-overlapping fields, and the number of cells that had traversed the membrane was counted using ImageJ software. The numbers of migrated and invaded cells were used to quantify migration and invasion, respectively. Each experiment was independently repeated at least three times. Representative microscopic images include a 200 μm scale bar.

### 2.10. Quantitative Real-Time PCR Analysis

Quantitative real-time PCR (qPCR) was performed to measure the mRNA expression levels of EMT-related genes and candidate BA-related molecules in PC-3 cells [48]. Cells were divided into four groups: control, BA alone (50 μM), TGF-β1 alone (5 ng/mL), and TGF-β1 + BA (5 ng/mL TGF-β1 + 50 μM BA). All groups were treated for 48 h.

Total RNA was extracted using an RNA extraction kit (CWBIO, Beijing, China; Cat. No. CW3705S-32). Genomic DNA was removed, and reverse transcription was performed using HiScript III All-in-one RT SuperMix Perfect for qPCR (Vazyme, Nanjing, China; Cat. No. R333-C1). qPCR was performed using SupRealQ Purple Universal SYBR qPCR Master Mix (Vazyme, Nanjing, China; Cat. No. Q412). Each 20 μL reaction contained 10 μL of 2× SYBR qPCR Master Mix, 0.4 μL of forward primer, 0.4 μL of reverse primer, 2 μL of cDNA template, and 7.2 μL of nuclease-free water. The amplification programme consisted of 37 °C for 2 min and 95 °C for 30 s, followed by 40 cycles of 95 °C for 10 s and 60 °C for 30 s. Melting curve analysis was performed after amplification.

GAPDH was used as the internal reference gene, and relative mRNA expression levels were calculated using the 2^^−ΔΔCt^ method [49]. The genes analysed included CDH1, VIM, SNAI1, ZEB1, DPP4, MME, and ABL1. Primer sequences are listed in Table 1. Each experiment was independently repeated at least three times.

### 2.11. Western Blot Analysis

Western blotting was performed to assess the expression levels of EMT-related proteins, candidate BA-related proteins, and associated signalling proteins in PC-3 cells following BA treatment [50]. Two experimental settings were used to examine BA effects: a BA concentration gradient and TGF-β1-induced treatment. In the BA concentration gradient experiment, cells were assigned to an untreated control group (Control) or BA-treated groups (20, 50, and 80 μM). In the TGF-β1-induced experiment, the groups included an untreated control group (Control), BA (50 μM), TGF-β1 (5 ng/mL), and TGF-β1 + BA (5 ng/mL TGF-β1 + 50 μM BA). The treatment duration was 48 h for all groups.

After treatment, total cellular proteins were extracted and quantified. Protein samples (15 μg) were separated by SDS-PAGE and transferred onto polyvinylidene fluoride (PVDF) membranes. After blocking, the membranes were incubated with the corresponding primary antibodies, followed by horseradish peroxidase (HRP)-conjugated secondary antibodies. Protein signals were detected using enhanced chemiluminescence (ECL). The proteins analysed included E-cadherin, N-cadherin, Snail, ZEB1, DPP4, MME, ABL1, TAZ, total AKT, and total ERK1/2, which were used to assess BA-related changes. Detailed antibody information is provided in Supplementary Table S1.

Band intensities were quantified using ImageJ software and normalised to GAPDH. Each experiment was independently repeated at least three times, and quantitative results were statistically analysed using independent biological replicates. The original uncropped Western blot images are provided in the Supplementary Materials.

### 2.12. Exploratory Transcriptomic Analysis

To characterise transcriptomic changes in PC-3 cells following BA treatment, we compared a control group with a BA-treated group (50 μM for 48 h), using three independent biological replicates per group. Following total RNA extraction and quality assessment, mRNA enrichment and library preparation were performed. Paired-end sequencing (PE150) was conducted on the Illumina NovaSeq 6000 platform. Raw sequencing reads were quality-filtered using fastp and aligned to the human reference genome (hg19) using HISAT2 (version 2.2.1). Gene expression was quantified using StringTie. Differential expression analysis was performed on the read count matrix using DESeq2 (version 1.46.0), with an adjusted *p*-value (padj) < 0.05 and |log2 fold change| > 1 as the screening thresholds [51]. Sample correlation analysis and principal component analysis (PCA) were performed using the TPM matrix. Gene Ontology (GO) and Kyoto Encyclopedia of Genes and Genomes (KEGG) enrichment analyses of differentially expressed genes were conducted using the clusterProfiler package, with multiple-testing correction performed using the Benjamini–Hochberg method.

### 2.13. Exploratory DIA Proteomic Analysis

DIA-based proteomic analysis was performed under the same treatment conditions as the transcriptomic analysis, with a control group and a BA-treated group (50 μM for 48 h), each comprising three independent biological replicates. Cellular proteins were extracted, quantified using the BCA assay, reduced, alkylated, digested with trypsin, and desalted using C18 columns. DIA analysis was performed using a Vanquish Neo nano-liquid chromatography system coupled to an Orbitrap Astral high-resolution mass spectrometer. Raw data were processed using DIA-NN (v1.8.1) for protein identification and quantification against the UniProt Homo sapiens protein database, with the protein identification false discovery rate (FDR) controlled below 1% [52]. Protein abundances were compared between groups using a two-sided *t*-test. Proteins with *p* < 0.05 and a fold change > 1.5 or <1/1.5 were considered differentially abundant. GO and KEGG enrichment analyses were subsequently performed, with enrichment significance assessed using Fisher’s exact test. These transcriptomic and proteomic analyses were used to explore molecular changes and potential biological processes associated with BA treatment. They were not intended to establish direct molecular targets or a complete mechanism of action.

### 2.14. Integrated Transcriptomic and Proteomic Analysis

Differentially expressed genes from the transcriptomic analysis and differentially abundant proteins from the proteomic analysis were converted to standard gene symbols and matched accordingly. Molecules meeting the respective differential expression criteria at both the transcriptomic and proteomic levels were defined as overlapping differentially expressed molecules. Based on the direction of log2 fold change in each dataset, these molecules were classified as upregulated at both the RNA and protein levels, downregulated at both levels, or showing opposite directions of change. A four-quadrant plot was generated to visualise these patterns. Representative overlapping molecules were further visualised, and GO and KEGG enrichment results were integrated to characterise the major molecular changes following BA treatment.

### 2.15. Statistical Analysis

Unless otherwise stated, all cell-based experiments were independently repeated at least three times. Quantitative data are presented as the mean ± standard deviation (SD). Sample sizes, numbers of technical replicates, and significance indicators are specified in the corresponding methods sections or figure legends. Comparisons between two groups were performed using an unpaired two-tailed Student’s *t*-test. Comparisons among multiple treatment groups at a single time point were performed using one-way analysis of variance (ANOVA), followed by Tukey’s multiple comparisons test. MTT assay data were analysed using two-way ANOVA, whereas wound-healing assay data were analysed using two-way repeated-measures ANOVA, followed by the appropriate multiple comparisons tests. A *p*-value < 0.05 was considered statistically significant.

Statistical analyses and graph generation for the cell-based experiments were performed using GraphPad Prism 9.5. Public transcriptomic data processing and related bioinformatics analyses were conducted in R version 4.4.3. The specific analytical methods, screening thresholds, and principal software packages are described in the corresponding methods sections.

The mass spectrometry proteomics data have been deposited to the ProteomeXchange Consortium via the PRIDE repository with the dataset identifier PXD085656 and DOI 10.6019/PXD085656.

## 3. Results

### 3.1. Screening and Machine Learning Prioritization of β-Boswellic-Acid-Related Candidate Molecules

PCA revealed distinct clustering patterns among the datasets before batch correction. After ComBat correction, batch effects were markedly reduced (Figure 1A,B). Differential expression analysis of the corrected discovery cohort identified 609 differentially expressed genes, including 362 upregulated and 247 downregulated genes, using FDR < 0.05 and |log2FC| > 0.585 as the screening thresholds. Intersecting these genes with 368 predicted BA-related molecules from SwissTargetPrediction and PharmMapper identified 22 candidate BA-related molecules for subsequent machine learning analyses.

A total of 127 candidate models were constructed using the expression profiles of these 22 BA-related molecules. Among the models evaluated, glmBoost + Elastic Net (alpha = 0.2) showed favourable overall performance, with an AUC of 0.937 in the discovery cohort. The AUC values in the three external validation cohorts, GSE46602, GSE55945, and GSE200879, were 0.980, 0.954, and 0.875, respectively, with an AUC of 0.942 in the combined external validation cohort (Figure 1C–E). The features retained by this model were further evaluated using Random Forest and SHAP analyses.

The Random Forest model achieved an AUC of 0.917 (Figure 1F). SHAP analysis showed that the top seven features accounted for approximately 80% of the total model contribution (Figure 1G). MME and ABL1 had relatively high mean absolute SHAP values, followed by candidate molecules including SRM, CRYZ, TGM3, and ABCC8 (Figure 1H–J). Based on the machine learning results, SHAP feature importance, and functional association network characteristics, DPP4, MME, and ABL1 were selected for further analysis.

### 3.2. Functional Enrichment and Association Characteristics of Candidate BA-Related Molecules

To further characterise the potential functional roles of candidate BA-related molecules, GO and KEGG enrichment analyses and GeneMANIA functional association network analysis were performed. GO enrichment analysis revealed that the candidate molecules were associated with various biological functions related to BA (Figure 2A). In the biological process (BP) category, the main enriched terms included cardiac muscle tissue growth, heart growth, organ growth, glucose metabolic process, regulation of extracellular matrix organisation, and polyamine biosynthetic process. The cellular component (CC) category mainly included endoplasmic reticulum lumen, focal adhesion, cell–substrate junction, and cytoplasmic side of plasma membrane. The molecular function (MF) category was primarily associated with NADP binding, peptide binding, carbohydrate binding, and nuclear receptor activity (Figure 2B,C).

KEGG enrichment analysis identified several pathways related to the candidate BA-related molecules, including Glutathione metabolism, Pentose phosphate pathway, Amino sugar and nucleotide sugar metabolism, Arginine and proline metabolism, Cysteine and methionine metabolism, Central carbon metabolism in cancer, PPAR signalling pathway, and ErbB signalling pathway (Figure 2D,E). These pathways were mainly associated with redox metabolism, nutrient and energy metabolism, and cancer-related signalling processes.

GeneMANIA functional association network analysis further revealed that DPP4, G6PD, ABL1, MME, and BCHE were functionally connected to multiple candidate molecules, with DPP4, G6PD, and ABL1 showing relatively more network connections (Figure 2F). Overall, the candidate BA-related molecules were mainly associated with metabolic regulation, extracellular matrix organisation, cell adhesion, and cancer-related signalling. These findings provided functional context for further investigating BA effects on migration, invasion, and EMT-related phenotypes in prostate cancer cells.

### 3.3. Molecular Docking and Molecular Dynamics Analysis of BA with Major Candidate Proteins

Based on candidate molecule screening, machine learning, and functional association analyses, DPP4, MME, and ABL1 were selected as proteins for molecular docking and molecular dynamics simulations with BA. Molecular docking revealed predicted binding energies of −9.8, −9.1, and −7.3 kcal/mol for the BA–DPP4, BA–MME, and BA–ABL1 complexes, respectively. BA–DPP4 showed the lowest predicted binding energy, followed by BA–MME and BA–ABL1 (Figure 3A,C,E).

Molecular dynamics simulations were subsequently performed for 100 ns on the three BA–protein complexes. RMSD analysis showed that all systems underwent varying degrees of conformational adjustment during the initial simulation period, followed by relatively stable fluctuations. More pronounced conformational changes were observed for BA–ABL1 during the early stages of simulation. RMSF analysis indicated that residue fluctuations were mainly concentrated in localised regions. The radius of gyration (Rg) and solvent-accessible surface area (SASA) of each BA–protein complex remained relatively stable throughout the simulations, without sustained directional changes. Hydrogen bond analysis showed that hydrogen bonds between BA and the corresponding proteins underwent dynamic formation and dissociation during the simulations (Figure 3B,D,F).

MM/PBSA analysis yielded binding free energies of −12.693, −7.851, and −8.739 kcal/mol for BA–DPP4, BA–MME, and BA–ABL1, respectively, with BA–DPP4 showing the lowest value. Taken together, the molecular docking, molecular dynamics, and binding free energy results indicated that BA–DPP4 exhibited relatively favourable predicted binding characteristics among the three tested complexes. BA also showed potential interactions with MME and ABL1. These computational findings provided preliminary support for potential molecular interactions between BA and the candidate proteins, although direct binding remains to be experimentally validated.

### 3.4. Effects of BA on PC-3 Cell Viability, Wound Closure, Migration, and Invasion

MTT assay results showed that higher concentrations of BA reduced PC-3 cell viability, with the inhibitory effect generally increasing with longer treatment durations (Figure 4A–F). The IC_50_ values of BA at 24, 48, and 72 h were 131.7, 109.7, and 73.85 μM, respectively. At lower concentrations, cell viability fluctuated without a consistent concentration-dependent decrease, whereas the inhibitory effects were more pronounced at higher concentrations.

The wound-healing assay showed that BA treatment (15, 25, and 35 μM) generally reduced wound closure in PC-3 cells compared with the control group, with more pronounced differences at 12 and 24 h and a trend towards a concentration-dependent effect (Figure 5A,B). The docetaxel group also showed reduced wound closure. TGF-β1 treatment promoted wound closure, and BA attenuated this effect when combined with TGF-β1.

Transwell assays showed that BA treatment generally reduced the number of migrated and invaded PC-3 cells, with more pronounced changes observed in the 35 μM BA group (Figure 6A,B). TGF-β1 treatment increased cell migration and invasion, and combined treatment with BA reduced the number of cells passing through the membrane compared with TGF-β1 treatment alone. Together, these results indicated that BA reduced PC-3 cell viability and suppressed migration- and invasion-related phenotypes.

### 3.5. Effects of BA on the Expression of EMT-Related Molecules and Candidate Proteins

qPCR analysis showed that TGF-β1 treatment decreased CDH1 expression and increased VIM, SNAI1, and ZEB1 expression, whereas BA (50 μM) treatment alone produced the opposite expression patterns. Compared with TGF-β1 treatment alone, combined treatment with BA increased CDH1 expression and decreased VIM, SNAI1, and ZEB1 expression, suggesting that BA attenuated TGF-β1-induced EMT-related transcriptional changes (Figure 7D–G). Among the candidate molecules, TGF-β1 treatment decreased MME and DPP4 expression but increased ABL1 expression, whereas BA treatment alone produced the opposite trends. Compared with TGF-β1 treatment alone, combined treatment with BA increased MME and DPP4 expression and decreased ABL1 expression (Figure 7A–C).

Western blot analysis showed that E-cadherin protein levels generally increased, whereas N-cadherin, Snail, and ZEB1 protein levels generally decreased across BA concentrations of 20–80 μM (Figure 8A,C). TGF-β1 treatment decreased E-cadherin levels and increased N-cadherin, Snail, and ZEB1 levels, while these changes were attenuated by combined treatment with BA (Figure 8A,C). DPP4, MME, and ABL1 protein levels generally decreased with increasing BA concentrations and were also lower following combined treatment with BA than following TGF-β1 treatment alone (Figure 8B). Notably, the changes in DPP4 and MME protein expression were not consistent with their corresponding mRNA levels.

In addition, total AKT, total ERK1/2, and TAZ protein levels generally decreased with increasing BA concentrations. Their levels increased following TGF-β1 treatment but were reduced by combined treatment with BA (Figure 8D). Because AKT and ERK phosphorylation levels were not measured, these findings reflect changes in total protein levels only. They cannot establish whether the corresponding signalling pathways were activated or inhibited.

### 3.6. Integrated Transcriptomic and Proteomic Analysis of BA-Induced Molecular Changes

Integrated transcriptomic and proteomic analyses investigated molecular changes in PC-3 cells following BA treatment. After gene-symbol deduplication, transcriptomic analysis identified 1267 differentially expressed genes, including 570 upregulated and 697 downregulated genes (Figure 9A). Proteomic analysis quantified 8564 protein entries and, after gene-symbol deduplication, identified 648 genes corresponding to differentially abundant proteins, with 187 increased and 461 decreased protein abundance (Figure 9B).

Intersection analysis identified 226 genes meeting the differential criteria in both datasets (Figure 9D). Of these, 83 were upregulated at both the RNA and protein levels, 142 were downregulated at both levels, and only one was downregulated at the RNA level but upregulated at the protein level, indicating predominantly concordant changes among the overlapping genes (Figure 9C). Protein abundance patterns of representative overlapping genes, including SMAD3, EPB41L5, HPSE, MYLK, TSPAN8, OCLN, LCN2, and ROR1, are shown in Figure 9E.

Functional enrichment analyses implicated biological processes and pathways related to cell cycle regulation, DNA replication, chromosome segregation, and DNA repair in both datasets (Figure 9F–I). The TGF-β signaling pathway was also among the 30 pathways with the lowest adjusted *p*-values in the transcriptomic KEGG analysis (adjusted *p* = 0.120; Figure 9G). Together, these results support BA-associated changes in gene and protein expression as a plausible molecular basis for the observed EMT-related phenotypes, migration, and invasion.

## 4. Discussion

This study evaluated the potential effects of BA in prostate cancer using in vitro phenotypic assays, candidate molecule screening, and multi-omics analyses. Overall, the findings suggested that BA affected migration, invasion, and EMT-related phenotypes in PC-3 cells, accompanied by molecular expression changes at multiple levels. These findings extend previous research on boswellic acids, which has largely focused on their antiproliferative and pro-apoptotic effects, and are broadly consistent with their reported antitumour potential [53]. EMT is closely associated with prostate cancer invasion, metastasis, and treatment resistance, with TGF-β acting as an important regulator [15,54,55,56,57]. In this study, BA treatment was associated with changes in EMT-related molecules, including E-cadherin, N-cadherin, Snail, and ZEB1, and attenuated TGF-β1-induced changes in migration, invasion, and EMT-related markers. Notably, the BA concentrations used in the migration and invasion assays were below the IC_50_ determined by the 48 h MTT assay. Therefore, the observed phenotypic changes were unlikely to be explained entirely by marked reductions in cell viability. However, the MTT assay primarily reflects cellular metabolic activity [58], and wound-healing assays may also be influenced by cell proliferation [46]. Thus, the present findings support a modulatory effect of BA on EMT-related migration and invasion rather than demonstrating complete reversal of EMT. Figure 10 summarises the effects of BA on TGF-β1-induced EMT-related molecular changes and the migration and invasion of PC-3 cells.

DPP4, MME, and ABL1 were prioritised as candidate BA-related molecules in this study. Previous studies have suggested that MME exerts tumour-suppressive effects in prostate cancer [59,60], whereas aberrant DPP4 expression has been associated with prostate cancer progression and castration resistance [61,62]. ABL family kinases are involved in the regulation of cell migration, invasion, and EMT, although their specific roles are context-dependent [63,64,65]. Molecular docking and molecular dynamics simulations were further performed to evaluate potential interactions between BA and these candidate proteins. However, such computational analyses provide only predictive evidence of molecular interactions. They cannot replace direct binding experiments or establish causal relationships. Therefore, DPP4, MME, and ABL1 should be regarded as candidate molecules associated with BA activity in this study rather than confirmed direct targets of BA.

Notably, DPP4 and MME showed inconsistent changes at the mRNA and protein levels following BA treatment, suggesting possible post-transcriptional regulation. mRNA abundance does not always correspond to protein abundance, as translational efficiency, protein stability, and degradation can contribute to discrepancies between the two [66,67,68]. The opposing expression patterns observed in this study may therefore involve compensatory transcriptional responses or regulation at the protein level, although the underlying mechanisms require further investigation. The current findings do not establish the specific functional roles of DPP4 or MME in the effects of BA. In addition, TGF-β-driven EMT can interact with signalling networks such as PI3K/AKT and MAPK [69,70,71], while the Hippo–YAP/TAZ axis is also involved in tumour progression and the regulation of cellular phenotypes [72,73,74,75]. Changes in total AKT, total ERK1/2, and TAZ protein levels were observed in this study. However, because AKT and ERK phosphorylation were not assessed, these findings cannot establish the activation or inhibition of the PI3K/AKT or MAPK/ERK signalling pathways. Integrated transcriptomic and proteomic analyses further showed that BA treatment was associated with molecular changes involving cell adhesion, cytoskeletal remodelling, cell cycle regulation, DNA replication, and TGF-β-related processes. These findings suggest that the effects of BA may extend beyond EMT-related changes and involve broader alterations in cellular states. Therefore, the multi-omics results provide exploratory insights into potential molecular processes associated with BA treatment but are insufficient to identify its direct regulatory pathways.

This study has several limitations. First, the in vitro experiments were conducted using only the PC-3 cell line, and the findings require validation in prostate cancer models with different molecular backgrounds. Second, functional perturbation and direct binding experiments involving DPP4, MME, and ABL1 were not performed, preventing the establishment of causal relationships between these candidate molecules and BA-induced phenotypic changes. In addition, the phosphorylation states of relevant signalling proteins were not assessed, and the sample sizes for the exploratory transcriptomic and proteomic analyses were relatively limited. The effective BA concentrations were relatively high in some experiments, while its in vivo exposure levels and bioavailability remain unclear. Animal experiments were also not conducted. Therefore, the in vivo effects of BA in prostate cancer and their molecular basis require further investigation using additional cell lines, functional perturbation studies, direct binding assays, and in vivo models.

## 5. Conclusions

In conclusion, BA suppressed migration and invasion in PC-3 cells and attenuated TGF-β1-induced EMT-related changes. Candidate molecules, including DPP4, MME, and ABL1, together with the multi-omics findings, offered exploratory insights into BA-associated molecular changes. However, the direct molecular targets and causal mechanisms underlying these effects require further validation.

## Figures and Tables

**Figure 1:**
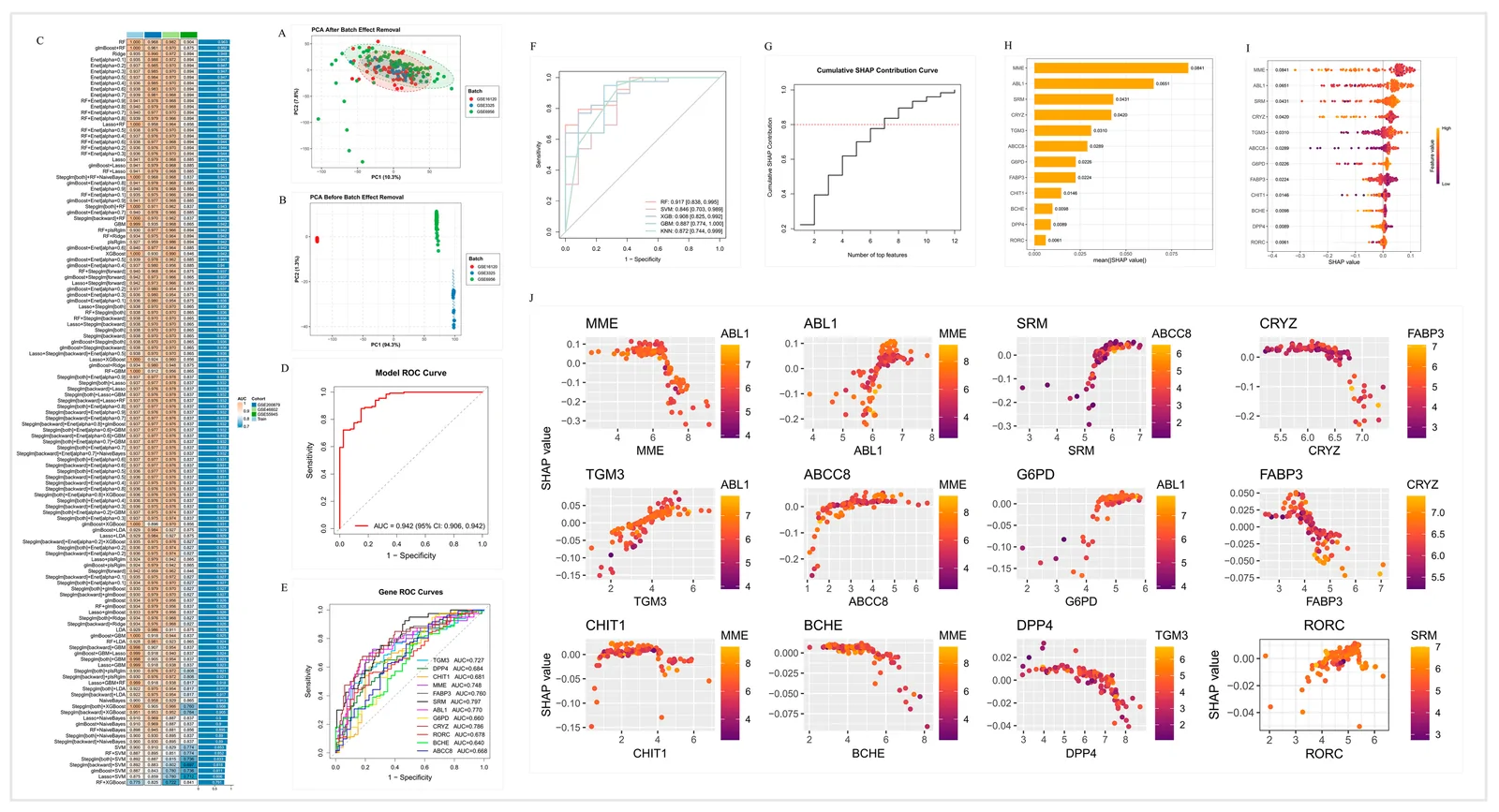
Screening and machine-learning-based prioritisation of candidate β-boswellic-acid-related molecules in prostate cancer. (**A**,**B**) PCA results for the discovery cohort after (**A**) and before (**B**) ComBat batch correction. (**C**) Comparison of AUC values across 127 candidate machine learning models in the discovery and external validation cohorts. (**D**) ROC curve of the glmBoost + Elastic Net model in the combined external validation cohort. (**E**) ROC curves for the features retained by the selected model. (**F**) ROC curves of RF, SVM, XGB, GBM, and KNN models constructed using the selected features. (**G**) Cumulative SHAP contribution curve based on feature importance, with the dashed line indicating the 80% cumulative contribution threshold. (**H**) Feature importance ranking based on mean absolute SHAP values. (**I**) SHAP summary plot showing the magnitude and direction of each feature’s contribution to the model output. (**J**) SHAP dependence plots showing the relationships between feature values and their corresponding SHAP values, with colours indicating the values of interacting features. The discovery cohort included 40 non-tumour control samples and 133 prostate cancer samples, while the three external validation cohorts included 31 non-tumour control samples and 177 prostate cancer samples. Model performance was evaluated using stratified five-fold cross-validation. Abbreviations: BA, β-boswellic acid; PCA, principal component analysis; AUC, area under the receiver operating characteristic curve; ROC, receiver operating characteristic; RF, random forest; SVM, support vector machine; XGB, eXtreme Gradient Boosting; GBM, gradient boosting machine; KNN, k-nearest neighbours; SHAP, SHapley Additive exPlanations.

**Figure 2:**
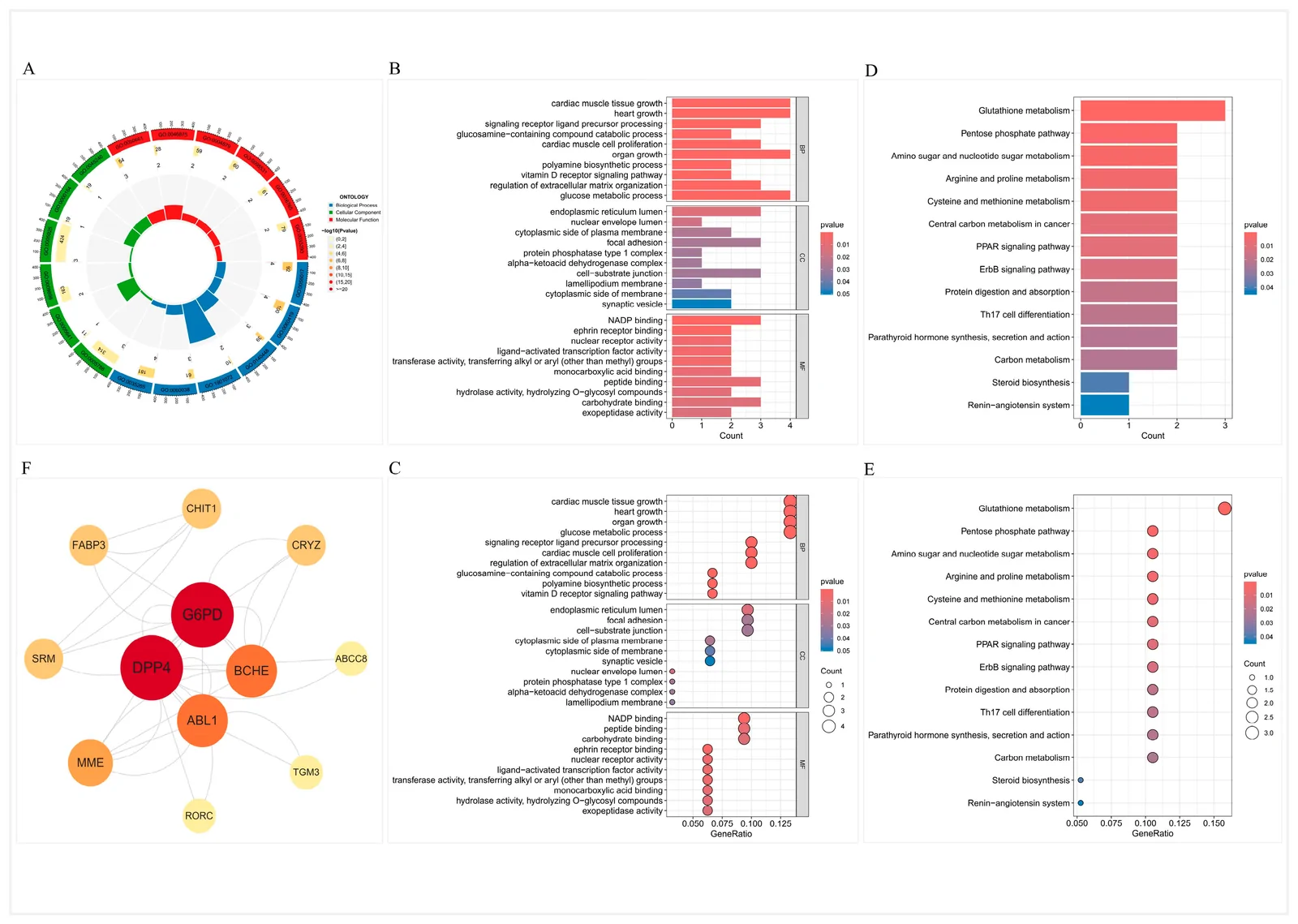
Functional enrichment and functional association network analyses of β-boswellic-acid-related candidate molecules. (**A**) Circular plot of Gene Ontology (GO) enrichment analysis of candidate BA-related molecules. (**B**) GO enrichment bar plot showing the main enriched terms in the biological process (BP), cellular component (CC), and molecular function (MF) categories. (**C**) GO enrichment bubble plot showing the GeneRatio, gene count, and enrichment significance of the main enriched terms. (**D**) Bar plot of Kyoto Encyclopedia of Genes and Genomes (KEGG) pathway enrichment analysis. (**E**) KEGG enrichment bubble plot showing the GeneRatio, gene count, and enrichment significance of the main enriched pathways. (**F**) Functional association network of candidate BA-related molecules constructed using GeneMANIA. GeneRatio represents the proportion of input genes associated with a given enriched term. Abbreviations: BA, β-boswellic acid; GO, Gene Ontology; BP, biological process; CC, cellular component; MF, molecular function; KEGG, Kyoto Encyclopedia of Genes and Genomes.

**Figure 3:**
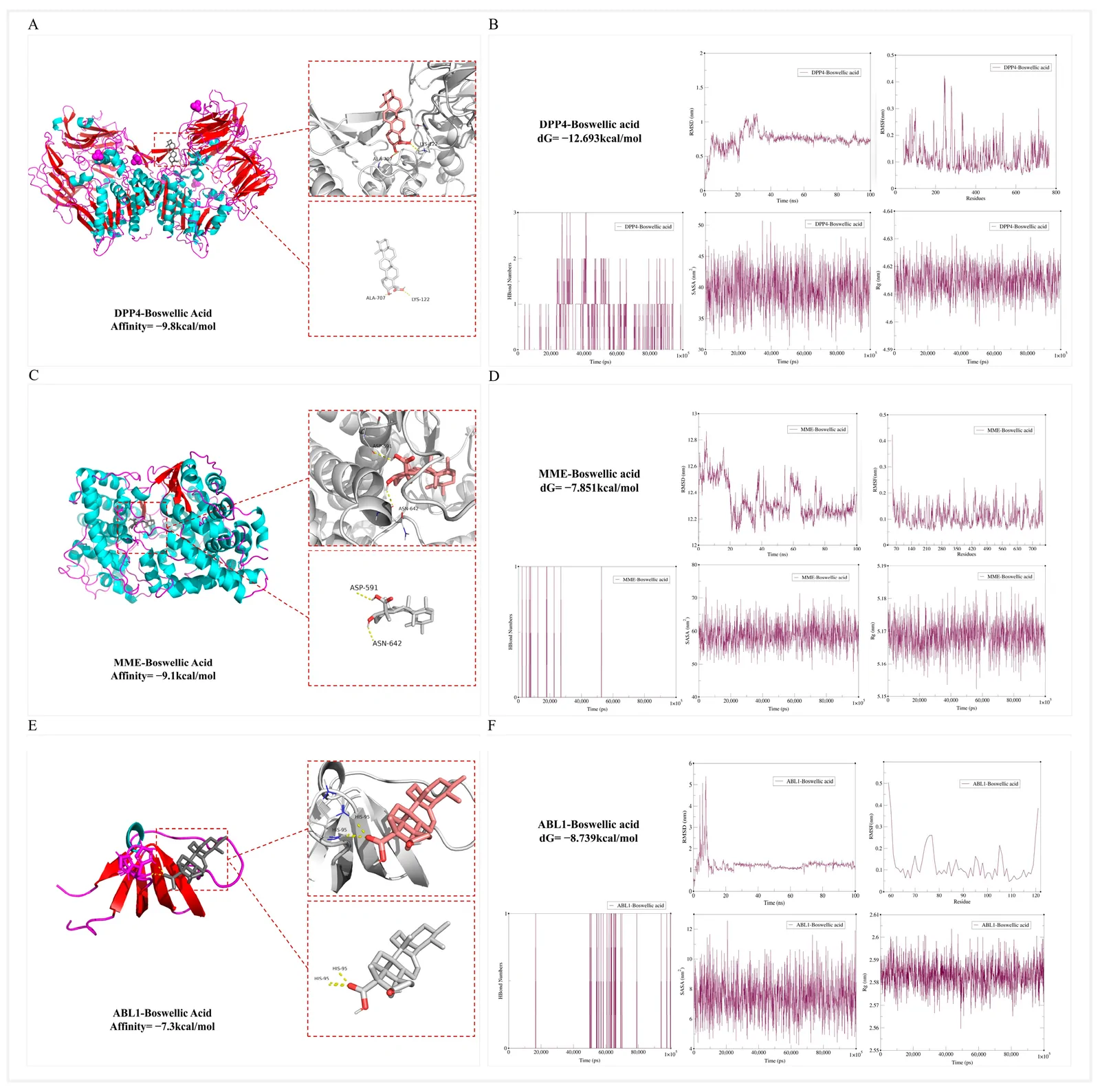
Molecular docking and molecular dynamics analyses of BA with DPP4, MME, and ABL1. (**A**) Molecular docking conformation and local interactions of BA–DPP4, with a predicted binding energy of −9.8 kcal/mol. (**B**) Results of the 100 ns molecular dynamics simulations of the BA–DPP4 complex, including RMSD, RMSF, number of hydrogen bonds, Rg, and SASA. (**C**) Molecular docking conformation and local interactions of BA–MME, with a predicted binding energy of −9.1 kcal/mol. (**D**) Results of the 100 ns molecular dynamics simulations of the BA–MME complex. (**E**) Molecular docking conformation and local interactions of BA–ABL1, with a predicted binding energy of −7.3 kcal/mol. (**F**) Results of the 100 ns molecular dynamics simulations of the BA–ABL1 complex. Three independent 100 ns molecular dynamics simulations were performed for each complex. MM/PBSA calculations yielded binding free energies of −12.693, −7.851, and −8.739 kcal/mol for BA–DPP4, BA–MME, and BA–ABL1, respectively. Abbreviations: BA, β-boswellic acid; DPP4, dipeptidyl peptidase 4; MME, membrane metalloendopeptidase; ABL1, ABL proto-oncogene 1, non-receptor tyrosine kinase; RMSD, root-mean-square deviation; RMSF, root-mean-square fluctuation; Rg, radius of gyration; SASA, solvent-accessible surface area; MM/PBSA, molecular mechanics/Poisson–Boltzmann surface area.

**Figure 4:**
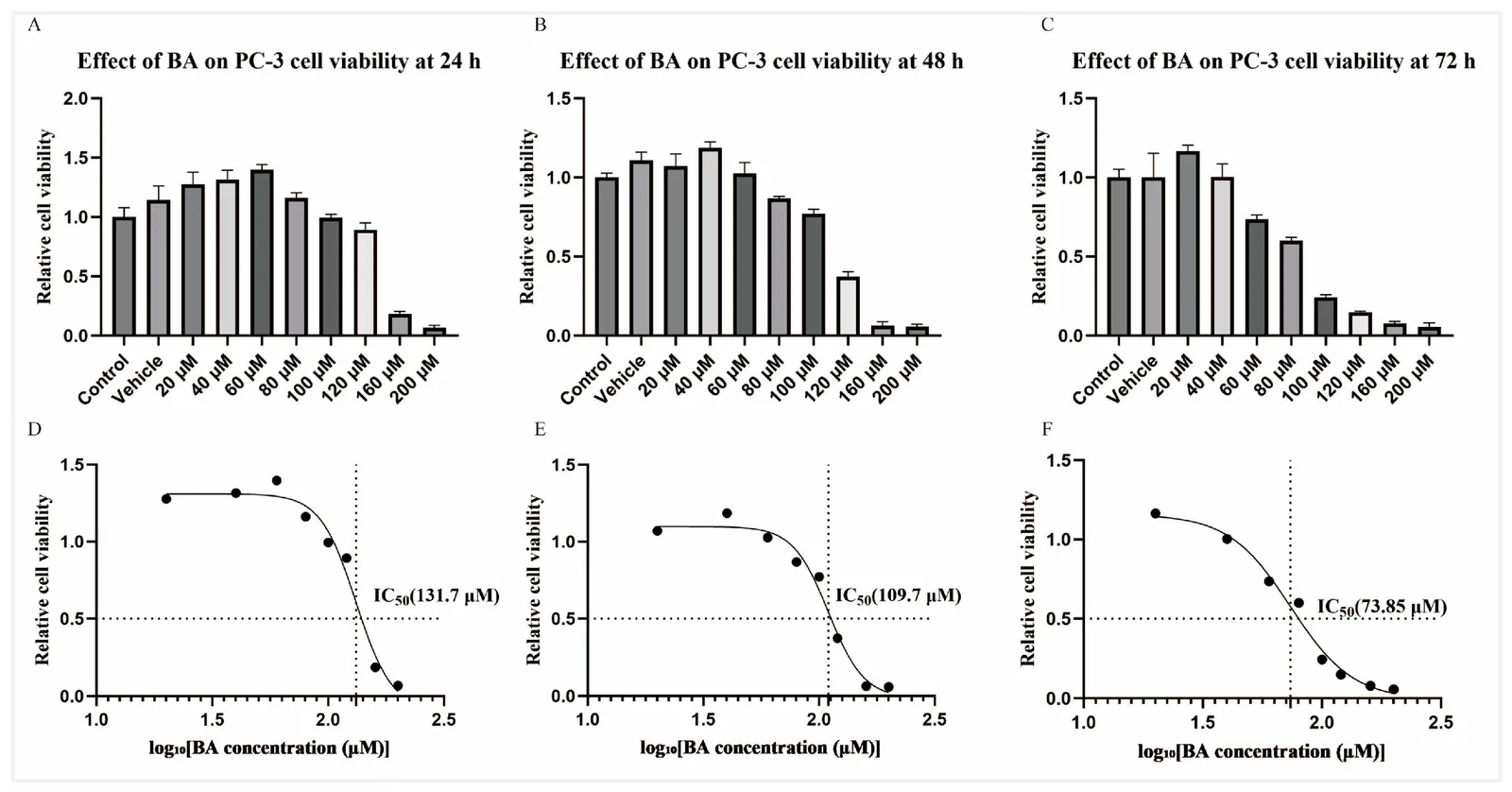
Effects of β-boswellic acid on PC-3 cell viability. (**A**–**C**) Relative viability of PC-3 cells after treatment with BA (20–200 μM) for 24, 48, and 72 h, as assessed by the MTT assay. (**D**–**F**) BA concentration–response curves generated by nonlinear regression analysis, with half-maximal inhibitory concentration (IC_50_) values of 131.7, 109.7, and 73.85 μM at 24, 48, and 72 h, respectively. The experimental groups included an untreated control group (Control), a vehicle control group (Vehicle), and BA-treated groups at different concentrations. Relative cell viability was normalised to the untreated control group. Bar graph data are presented as the mean ± standard deviation (SD) from at least three independent experiments. MTT data were analysed using two-way analysis of variance (ANOVA). Abbreviations: BA, β-boswellic acid; MTT, 3-(4,5-dimethylthiazol-2-yl)-2,5-diphenyltetrazolium bromide; IC_50_, half-maximal inhibitory concentration; SD, standard deviation; ANOVA, analysis of variance.

**Figure 5:**
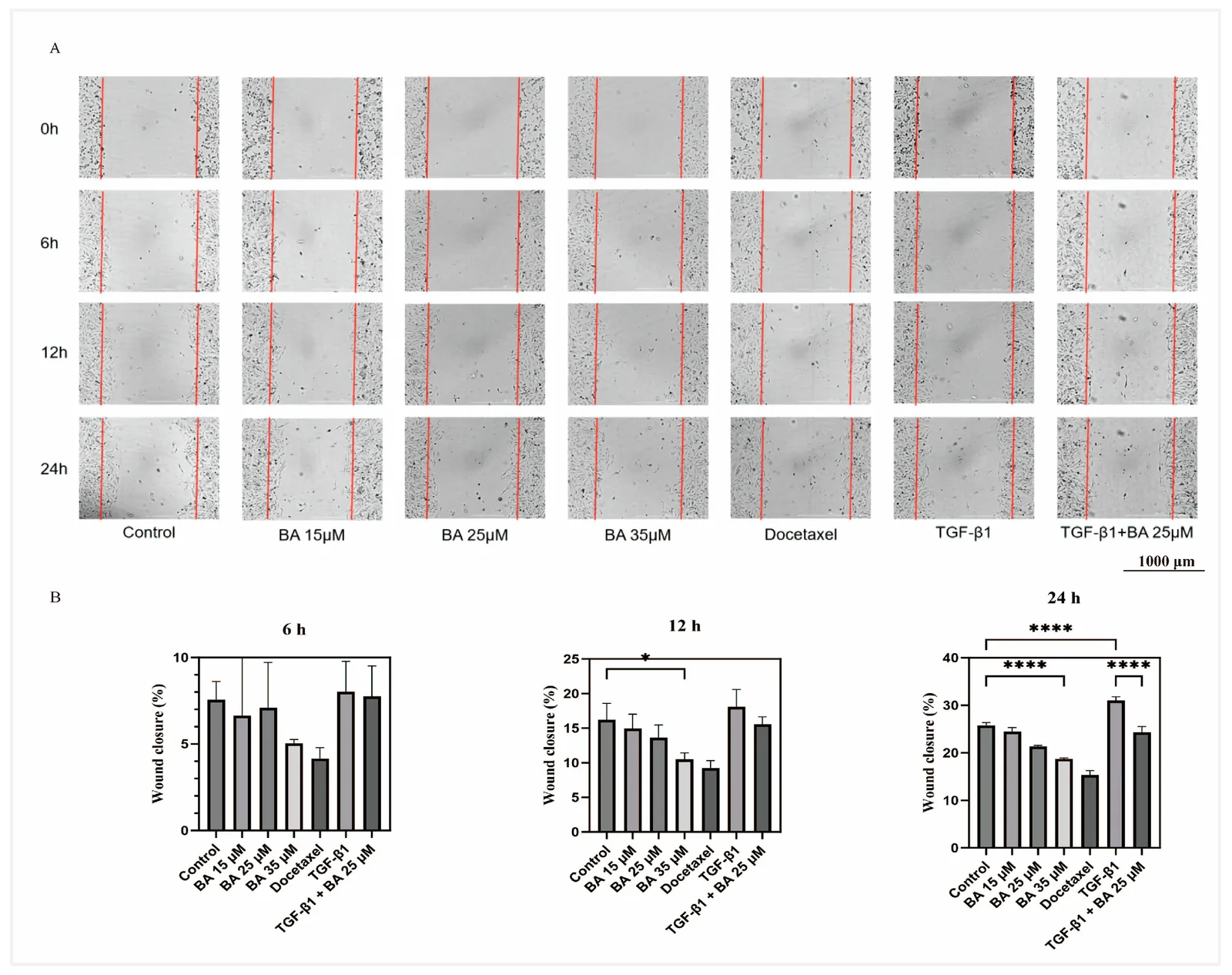
Effects of β-boswellic acid on wound closure in PC-3 cells. (**A**) Representative images of wound closure in PC-3 cells under different treatment conditions, captured at 0, 6, 12, and 24 h. The experimental groups included an untreated control group (Control), BA-treated groups (15, 25, and 35 μM), a docetaxel group (20 nM), a TGF-β1 group (5 ng/mL), and a TGF-β1 + BA group (5 ng/mL TGF-β1 + 25 μM BA). Scale bar = 1000 μm. (**B**) Quantitative analysis of wound closure rates at 6, 12, and 24 h across the treatment groups. Data are presented as the mean ± standard deviation (SD) from at least three independent experiments. Statistical analysis was performed using two-way repeated-measures ANOVA, followed by the appropriate multiple comparisons tests. Statistical significance is indicated as follows: * *p* < 0.05; **** *p* < 0.0001. Abbreviations: BA, β-boswellic acid; TGF-β1, transforming growth factor-beta 1; SD, standard deviation; ANOVA, analysis of variance.

**Figure 6:**
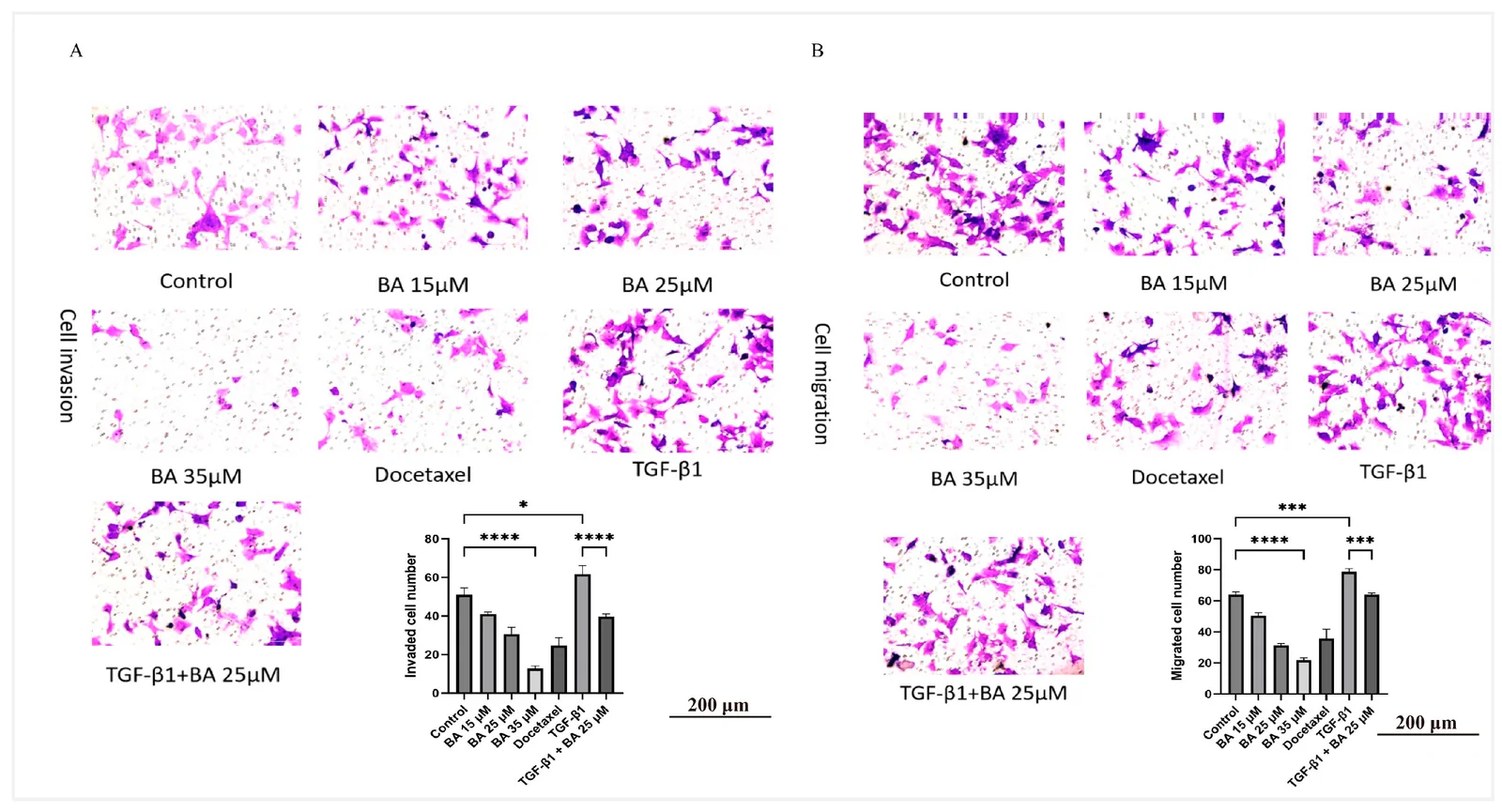
Effects of β-boswellic acid on PC-3 cell migration and invasion. (**A**) Representative Transwell images and quantitative analysis of invaded PC-3 cells under different treatment conditions. (**B**) Representative Transwell images and quantitative analysis of migrated PC-3 cells under different treatment conditions. The experimental groups included an untreated control group (Control), BA-treated groups (15, 25, and 35 μM), a docetaxel group (20 nM), a TGF-β1 group (5 ng/mL), and a TGF-β1 + BA group (5 ng/mL TGF-β1 + 25 μM BA). The treatment duration was 24 h. Scale bar = 200 μm. Quantitative data are presented as the mean ± standard deviation (SD) from at least three independent experiments. Multiple-group comparisons were performed using one-way ANOVA, followed by Tukey’s multiple comparisons test. Statistical significance is indicated as follows: * *p* < 0.05; *** *p* < 0.001; **** *p* < 0.0001. Abbreviations: BA, β-boswellic acid; TGF-β1, transforming growth factor-beta 1; SD, standard deviation; ANOVA, analysis of variance.

**Figure 7:**
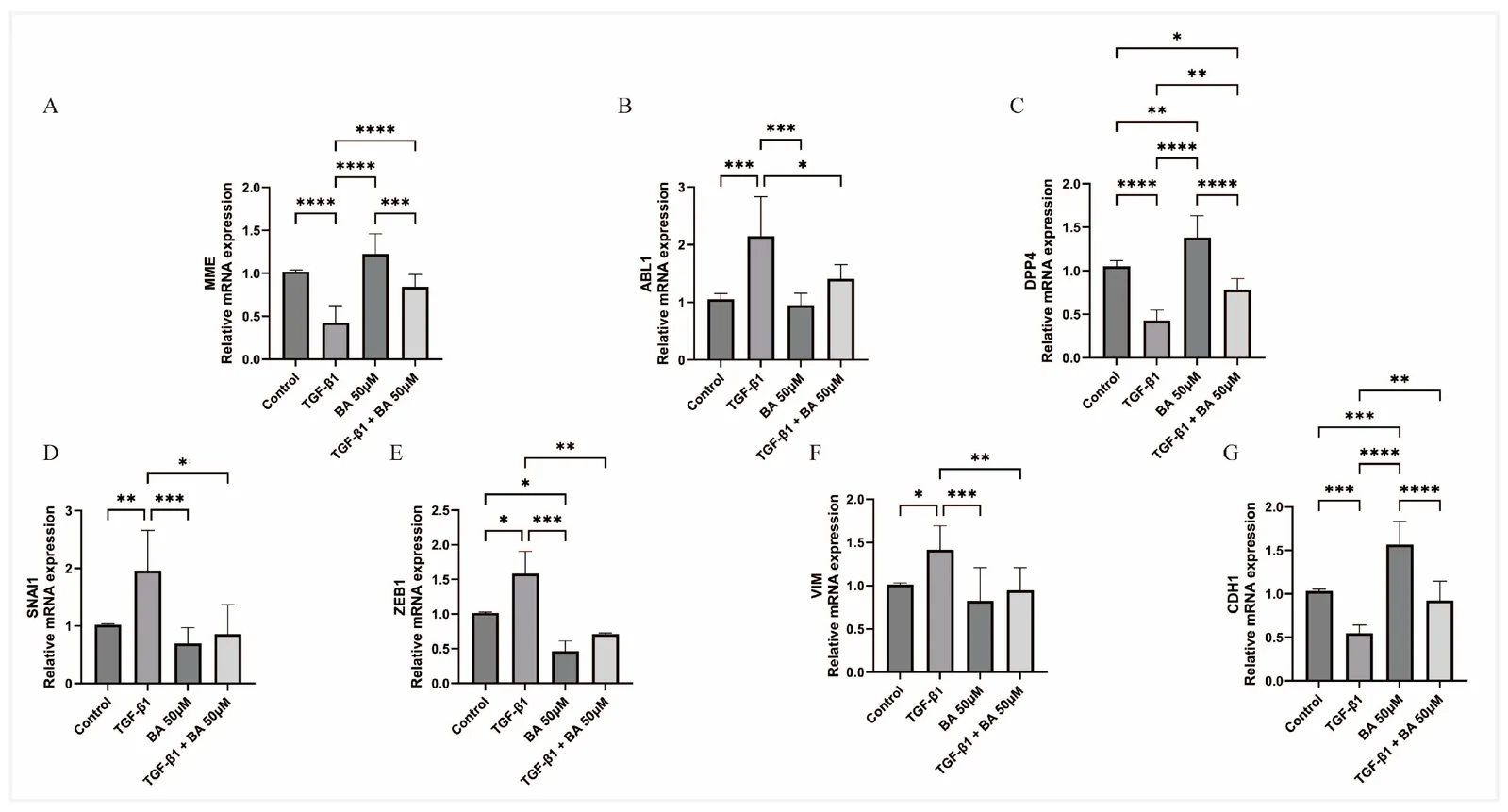
Effects of β-boswellic acid on the mRNA expression of EMT-related genes and candidate molecules in PC-3 cells. (**A**–**C**) Relative mRNA expression levels of MME, ABL1, and DPP4 under different treatment conditions, as determined by qPCR. (**D**–**G**) Relative mRNA expression levels of the EMT-related genes SNAI1, ZEB1, VIM, and CDH1, as determined by qPCR. The experimental groups included an untreated control group (Control), a BA-treated group (50 μM), a TGF-β1 group (5 ng/mL), and a TGF-β1 + BA group (5 ng/mL TGF-β1 + 50 μM BA). The experimental duration was 48 h for all groups. GAPDH was used as the internal reference gene, and relative mRNA expression levels were calculated using the 2^−ΔΔCt^ method. Data are presented as the mean ± standard deviation (SD) from at least three independent experiments. Multiple-group comparisons were performed using one-way ANOVA, followed by Tukey’s multiple comparisons test. Statistical significance is indicated as follows: * *p* < 0.05; ** *p* < 0.01; *** *p* < 0.001; **** *p* < 0.0001. Abbreviations: BA, β-boswellic acid; EMT, epithelial–mesenchymal transition; qPCR, quantitative real-time polymerase chain reaction; mRNA, messenger RNA; MME, membrane metalloendopeptidase; ABL1, ABL proto-oncogene 1, non-receptor tyrosine kinase; DPP4, dipeptidyl peptidase 4; TGF-β1, transforming growth factor-beta 1; GAPDH, glyceraldehyde-3-phosphate dehydrogenase; SD, standard deviation; ANOVA, analysis of variance.

**Figure 8:**
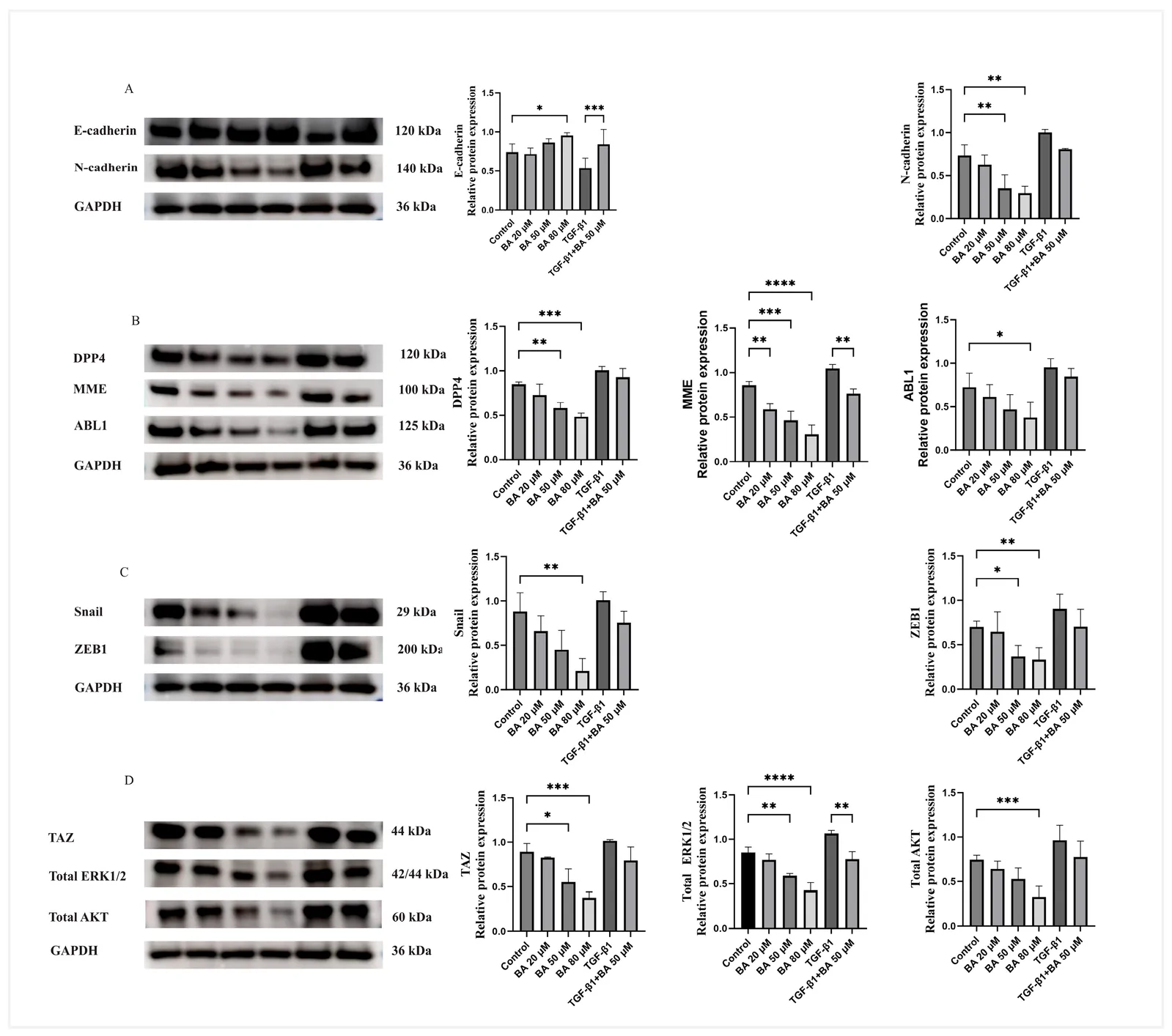
Effects of β-boswellic acid on the expression of EMT-related proteins, candidate proteins, and associated signalling proteins in PC-3 cells. (**A**) Representative Western blot images and quantitative analysis of E-cadherin and N-cadherin protein expression. (**B**) Representative Western blot images and quantitative analysis of DPP4, MME, and ABL1 protein expression. (**C**) Representative Western blot images and quantitative analysis of Snail and ZEB1 protein expression. (**D**) Representative Western blot images and quantitative analysis of TAZ, total ERK1/2, and total AKT protein expression. Two experimental settings were used. The BA concentration gradient experiment included an untreated control group (Control) and BA-treated groups (20, 50, and 80 μM). The TGF-β1-induced experiment included an untreated control group (Control), a BA-treated group (50 μM), a TGF-β1 group (5 ng/mL), and a TGF-β1 + BA group (5 ng/mL TGF-β1 + 50 μM BA). The experimental duration was 48 h for all groups. Protein expression levels were normalised to GAPDH. Quantitative data are presented as the mean ± standard deviation (SD) from at least three independent experiments. Multiple-group comparisons were performed using one-way analysis of variance (ANOVA), followed by Tukey’s multiple comparisons test. Statistical significance is indicated as follows: * *p* < 0.05; ** *p* < 0.01; *** *p* < 0.001; **** *p* < 0.0001. Abbreviations: BA, β-boswellic acid; EMT, epithelial–mesenchymal transition; DPP4, dipeptidyl peptidase 4; MME, membrane metalloendopeptidase; ABL1, ABL proto-oncogene 1, non-receptor tyrosine kinase; TAZ, transcriptional coactivator with PDZ-binding motif; ERK, extracellular signal-regulated kinase; AKT, protein kinase B; TGF-β1, transforming growth factor-beta 1; GAPDH, glyceraldehyde-3-phosphate dehydrogenase; SD, standard deviation; ANOVA, analysis of variance.

**Figure 9:**
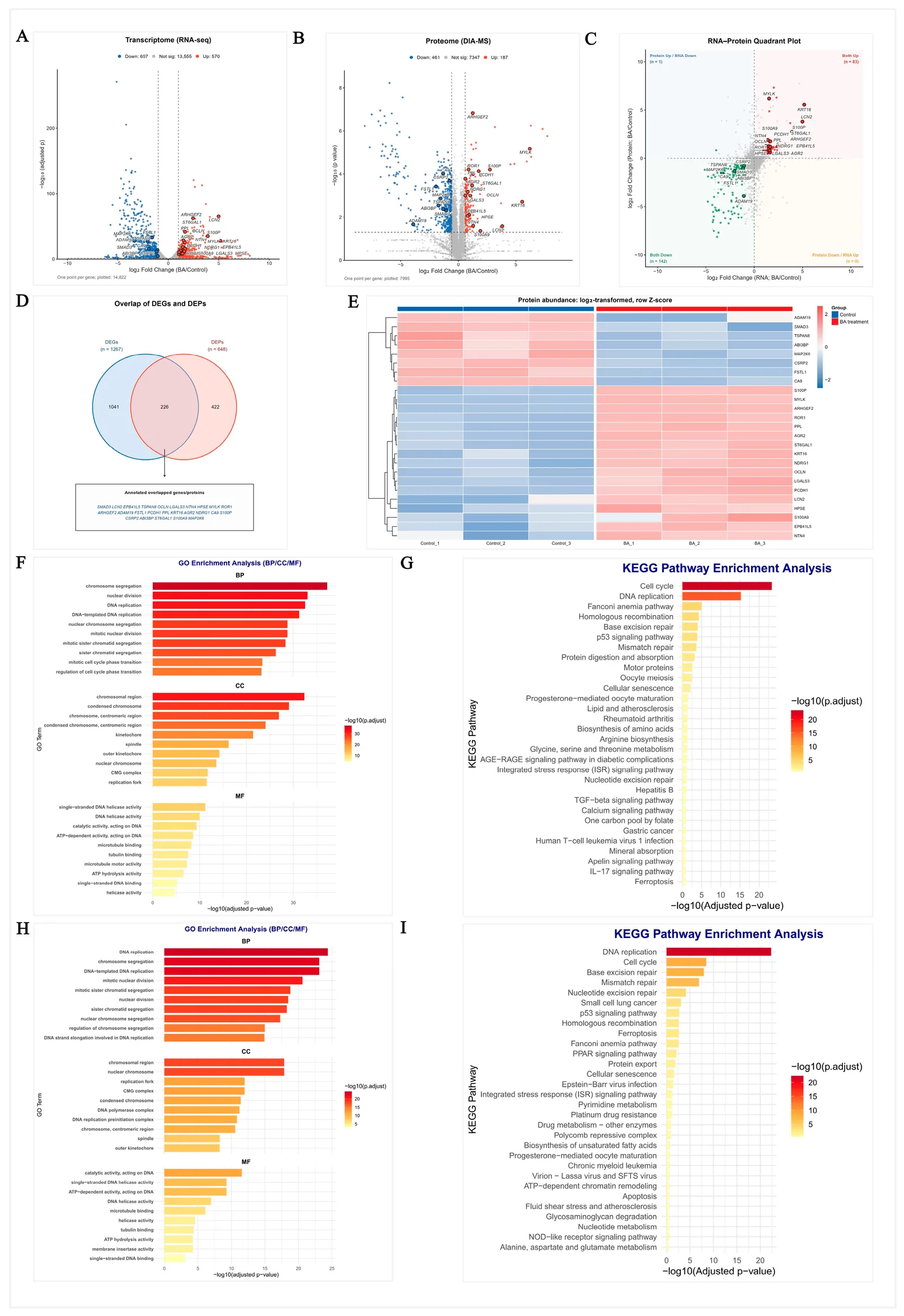
Integrated transcriptomic and proteomic analyses of PC-3 cells following BA treatment. (**A**) Transcriptomic volcano plot. (**B**) Proteomic volcano plot. In A and B, records were deduplicated by gene symbol, with one representative record retained per gene to ensure direct comparison. Fold changes are expressed as BA/Control. (**C**) Four-quadrant plot of RNA and protein fold changes. Coloured points represent genes meeting the respective differential criteria in both datasets; grey points represent the remaining genes. (**D**) Intersection of 1267 differentially expressed genes and 648 genes corresponding to differentially abundant proteins, identifying 226 overlapping differential genes. (**E**) Heatmap of protein abundances for representative overlapping genes, highlighting the shared regulation pattern. Protein abundances were log_2_-transformed and standardized to Z-scores within each row. Red and blue indicate higher and lower relative protein abundance, respectively. (**F**,**G**) GO and KEGG enrichment analyses of transcriptomic differentially expressed genes to assess functional changes. (**H**,**I**) GO and KEGG enrichment analyses of genes corresponding to differentially abundant proteins to compare protein-level functions. GO plots show the 10 terms with the lowest adjusted *p*-values in each of the BP, CC, and MF categories. KEGG plots show the 30 pathways with the lowest adjusted *p*-values, of which 16 in G and 14 in I met the adjusted *p* < 0.05 significance threshold. Bar lengths and colors represent −log_10_(adjusted p). Both omics analyses included an untreated control group (Control) and a BA-treated group (50 μM for 48 h), with three independent biological replicates per group.

**Figure 10:**
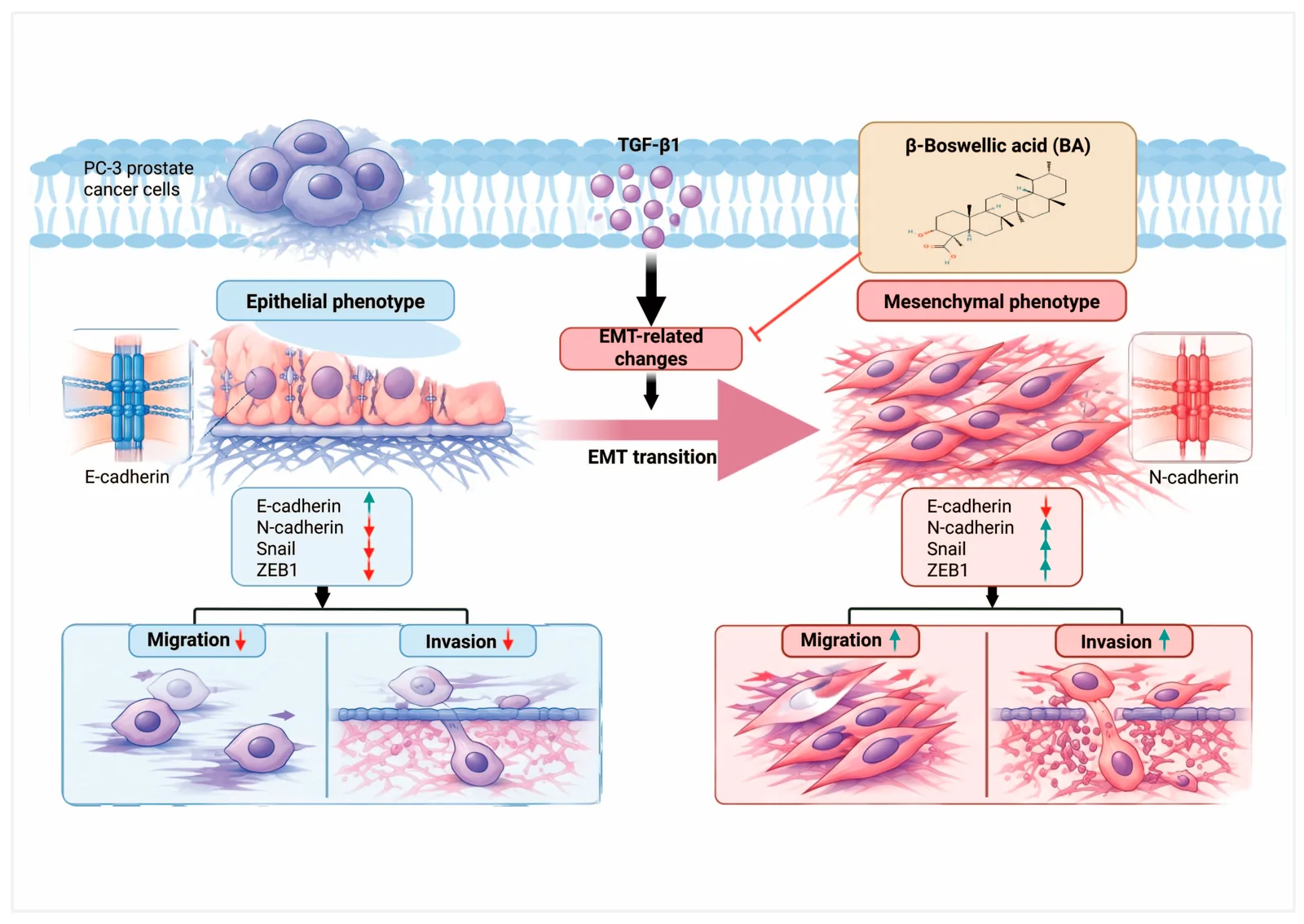
Schematic illustration of the effects of β-boswellic acid on TGF-β1-induced EMT-related changes and the migration and invasion of PC-3 cells. TGF-β1 treatment induced EMT-related changes in PC-3 cells, characterised by decreased E-cadherin expression, increased N-cadherin, Snail, and ZEB1 expression, and enhanced cell migration and invasion. BA co-treatment attenuated these changes, with increased E-cadherin expression, decreased N-cadherin, Snail, and ZEB1 expression, and reduced cell migration and invasion compared with TGF-β1 treatment alone. This schematic summarises the experimental findings of the present study and illustrates only the observed effects of BA, not a direct molecular mechanism. Abbreviations: BA, β-boswellic acid; TGF-β1, transforming growth factor-beta 1; EMT, epithelial–mesenchymal transition.

**Table 1:** Primer sequences of EMT-related and selected genes used for qPCR analysis.

Gene	Forward Primer (5′–3′)	Reverse Primer (5′–3′)
MME	CTTTAGTGCCCAGCAGTCCA	GAGTCCACCAGTCAACGAGG
ABL1	CAGGGAAGGCTGGAGGAAAG	TCGTGGCTTTTGTCTTTGCG
DPP4	ATGGGTCTCCCAACTCCAGA	TCCACTCCAACATCGACCAG
SNAI1	TCTGAGGCCAAGGATCTCCA	TGGCTTCGGATGTGCATCTT
ZEB1	TCTTCACCGTCTCTTTCAGCATCAC	GAGGAGAACTGGTTGCCTGTAATGG
VIM	AGGCAAAGCAGGAGTCCACTGA	ATCTGGCGTTCCAGGGACTCAT
CDH1	GGATGTGAATGAAGCCCCCA	GGATGTGATTTCCTGGCCCA
GAPDH	ACCACAGTCCATGCCATCAC	TTCCCGTTCAGCTCAGGGAT

## Data Availability

The publicly available datasets analyzed in this study are available from the Gene Expression Omnibus (GEO) database under accession numbers GSE3325, GSE6956, GSE16120, GSE46602, GSE55945, and GSE200879. The RNA-seq data generated in this study have been submitted to GEO and are currently being processed, with the accession number pending and to be added to the manuscript before publication. The mass spectrometry proteomics data have been deposited in the ProteomeXchange Consortium via the PRIDE repository under the dataset identifier PXD085656 (DOI: 10.6019/PXD085656).

## Supplementary Materials

The following supporting information can be downloaded at: https://www.mdpi.com/article/10.3390/cimb48101069/s1.

## Abbreviations

The following abbreviations are used in this manuscript:

BA	β-boswellic acid
ADT	androgen deprivation therapy
CRPC	castration-resistant prostate cancer
EMT	epithelial–mesenchymal transition
TGF-β1	transforming growth factor-β1
DMSO	dimethyl sulfoxide
GEO	Gene Expression Omnibus
PCA	principal component analysis
FDR	false discovery rate
AUC	area under the receiver operating characteristic curve
ROC	receiver operating characteristic
SVM	support vector machine
RF	random forest
GBM	gradient boosting machine
LDA	linear discriminant analysis
XGB	XGBoost
KNN	k-nearest neighbors
SHAP	SHapley Additive exPlanations
GO	Gene Ontology
BP	biological process
CC	cellular component
MF	molecular function
KEGG	Kyoto Encyclopedia of Genes and Genomes
PDB	Protein Data Bank
RMSD	root-mean-square deviation
RMSF	root-mean-square fluctuation
Rg	radius of gyration
SASA	solvent-accessible surface area
MM/PBSA	molecular mechanics/Poisson–Boltzmann surface area
NVT	constant-number, constant-volume, and constant-temperature ensemble
NPT	constant-number, constant-pressure, and constant-temperature ensemble
MTT	3-(4,5-dimethylthiazol-2-yl)-2,5-diphenyltetrazolium bromide
PBS	phosphate-buffered saline
qPCR	quantitative real-time PCR
GAPDH	glyceraldehyde-3-phosphate dehydrogenase
SDS-PAGE	sodium dodecyl sulfate–polyacrylamide gel electrophoresis
PVDF	polyvinylidene fluoride
HRP	horseradish peroxidase
ECL	enhanced chemiluminescence
DIA	data-independent acquisition
BCA	bicinchoninic acid
TPM	transcripts per million
ANOVA	analysis of variance
SD	standard deviation
IC_50_	half-maximal inhibitory concentration
